# Mass distribution and shape influence the perceived weight of objects

**DOI:** 10.3758/s13414-023-02780-8

**Published:** 2023-09-21

**Authors:** J. W. C. Harris, P. A. Chouinard

**Affiliations:** Department of Psychology, Counselling and Therapy, School of Psychology and Public Health, La Trobe University, Room 460, George Singer Building, Bundoora Campus, Melbourne, Victoria 3086 Australia

**Keywords:** Weight perception, Rotational dynamics, Mass distribution

## Abstract

Research suggests that the rotational dynamics of an object underpins our perception of its weight. We examine the generalisability of that account using a more ecologically valid way of manipulating an object’s mass distribution (mass concentrated either at the top, bottom, centre, near the edges or evenly distributed throughout the object), shape (cube or sphere), and lifting approach (lifting directly by the hand or indirectly using a handle or string). The results were in line with our predictions. An interaction effect was found where the mass distribution and lifting approach both associated with the lowest rotational dynamics made the stimulus appear lighter compared to other combinations. These findings demonstrate rotational dynamic effects in a more run-of-the-mill experience of weight perception than what has been demonstrated before using cumbersome stimuli.

## Introduction

In the early days of experimental psychology, Weber (1831) described the relationship between mass and perceived weight as being different, but systematic. However, mass in and of itself is unlikely to underlie our perception of weight as, for example, objects of the same mass but different sizes can be felt to be unequal in weight, as evidenced by the size-weight illusion (Charpentier, 1891). The illusion highlights that our ability to perceive weight is more nuanced than a simple parallel to a set of scales – it depends on more than just mass. Much previous work has considered the role of expectations in our perception of weight. While important, it is not the focus of the current work (e.g., Buckingham & Goodale, 2010; Dijker, 2008; Ellis & Lenerman, 1998; Flanagan et al., 2008; Ross, 1969). Instead, we consider the physical property of rotational dynamics.

The rotational dynamics account proposes that our perception of an object’s weight can be explained by the resistance to rotational forces that we experience in our limbs when we pick it up (Amazeen & Turvey, 1996). These forces are related to the object’s mass and how that mass is distributed within its volume. Broadly speaking, an object with greater rotational inertia has a greater resistance to being rotated and is perceived as heavier (Carello & Turvey, 2000). Because rotational inertia is related to both mass and mass distribution, it means that two objects with the same shape, mass, volume and density could differ in their rotational dynamics. As such, their perceived weight could change depending on how the mass is distributed within those objects (Koseleff, 1937; Wagman et al., 2007). The symmetry of an object’s rotational forces have also been reported to influence weight perception such that more rotationally symmetrical objects are perceived as lighter (Carello & Turvey, 2000). For example, a cube is less symmetrical than a sphere due to its corners. This means that the former, relative to the latter, requires more variable force to rotate it and consequently could convey a greater sense of weight. Therefore, with the same volume, mass and mass distribution, cubes could be perceived as heavier than spheres due to differences in rotational symmetry.

**Fig. 1:**
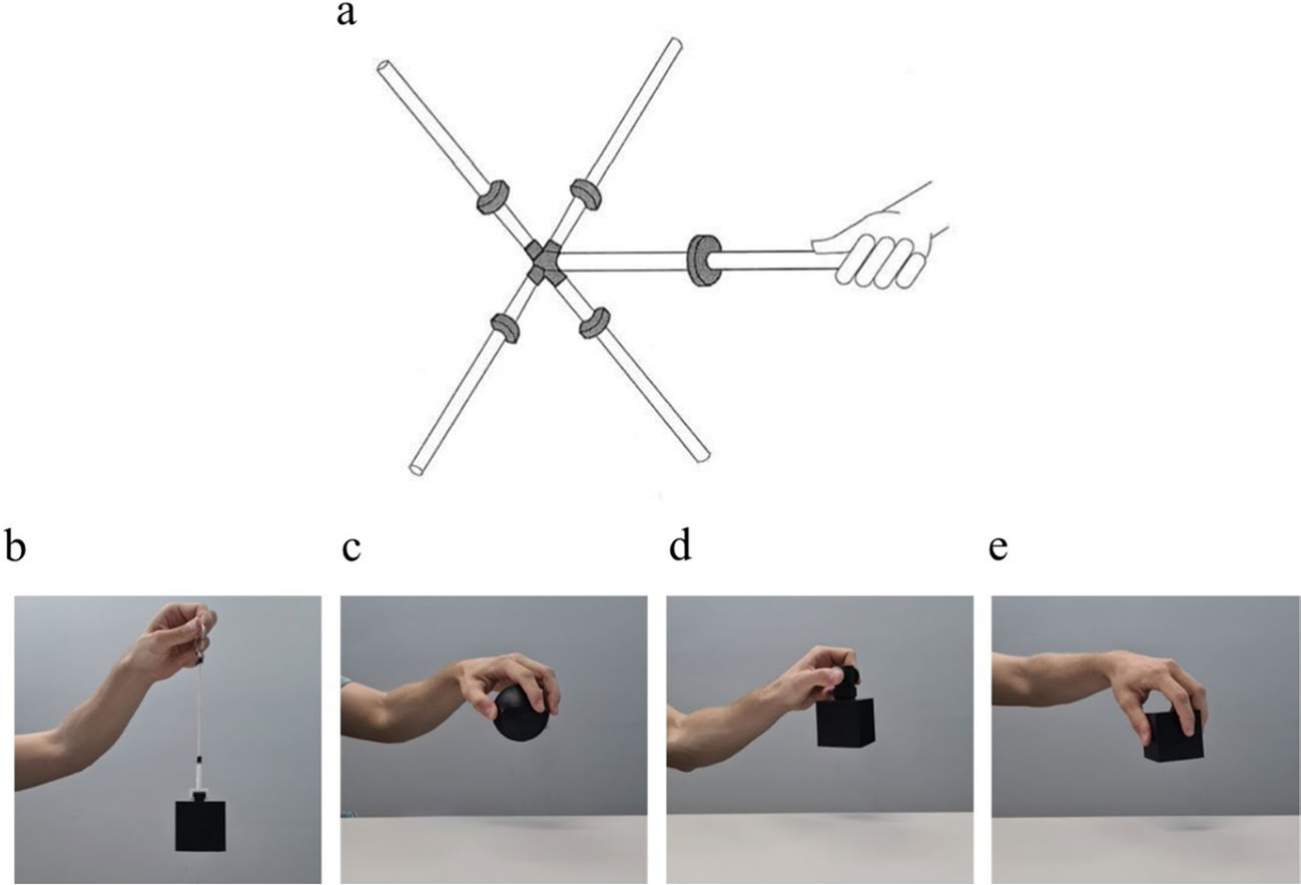
Tensor object and stimuli used in the current study. (**a**) Is an example tensor object used by Amazeen and Turvey (1996) to study inertia tensor. Each of the grey discs could be moved along the rods which allowed for the independent modification of each of the three eigenvalues of the inertia tensor. Subfigures (**b**) through (**e**) show the stimuli used in the current study being lifted

As weight perception is reportedly related to rotational dynamics, its effect could depend on our ability to reliably detect them (Burton et al., 1990). When objects are lifted in such a way as to minimise or eliminate one’s ability to feel the rotational forces of the object, for example with strings, weight perception should not be influenced by gross differences in them. Rather, weight perception should be more related to the influence of the object’s mass. With this in mind, Amazeen and Turvey (1996) tested their hypotheses with the use of a tensor object – an object that consisted of a cross attached at the end of a rod such that its four spokes were at a 90° angle with the central rod (see Fig. 1). Weights were then moved along the length of the cross spokes and the central rod to emulate desired eigenvalues. This rod, with its attached weights, was wielded by participants – being swung up and down with the wrist. The object’s construction and the wielding by the participants were done to ensure the participants could feel the rotational forces of the tensor object such that one could determine its influence on weight perception.

However, the explanation is purported to account for weight perception more broadly. Emulating the eigenvalues of standard stimuli, as was done in that study, may not necessarily be analogous to the more run-of-the-mill experiences we encounter with objects in our lives. Similarly, the lifting approach instructions given to the participants, while controlled and ensured that participants felt the rotational manipulations, is artificial and may not necessarily encapsulate naturalistic lifting behaviours. Yet this capacity to be able to detect differences in rotational forces in naturalistic objects lifted in an ecologically valid way would be necessary to claim rotational dynamics as an underpinning of weight perception for ordinary objects in the real world. The current study endeavoured to evaluate this.

Therefore, in the present investigation, we manipulated the mass distribution, lifting approach, and shape of objects to determine if the predicted rotational dynamics effects on weight perception can also be observed in a more ecologically valid setting. Specifically, we evaluated whether (1) mass distribution variations could influence the perceived weight of objects, (2) more rotationally symmetrical spheres could be perceived as lighter than cubes, and (3) the influence of an object’s mass distribution could diminish with non-direct (i.e., with string and handles) compared to direct (i.e., directly with the hand) approaches of lifting. In addition to determining if rotational dynamics has an influence on weight perception, we also sought in a separate experiment to evaluate if the experimental changes in mass distributions were noticeable to the participants.

## Methods

### Participants

A sample of 21 participants was collected; however, one was removed during the pre-processing process, as their scores in multiple conditions were 4 standard deviations (SDs) greater than our sample’s mean after normalising their data to the same scale as the other participants. Hence, the final sample was 20 participants (14 females and six males aged between 18 and 41 years, mean = 22.7 years). All participants were compensated with gift vouchers for their time. Participants reported being free of psychiatric, psychological and neurological disorders, as well as not currently taking medications that could make them drowsy. These exclusion criteria were in place so our sample reflected healthy brain functioning. All participants were also right-handed, as confirmed by the Flinders Handedness Survey (Nicholls et al., 2013), and had normal or corrected-to-normal vision, as verified by performance on the Snellen Chart and the Random Dot 3s Stereo Acuity Test (Vision Assessment Corporation, Illinois, USA).

### Stimuli and procedure

#### Experiment 1

**Fig. 2:**
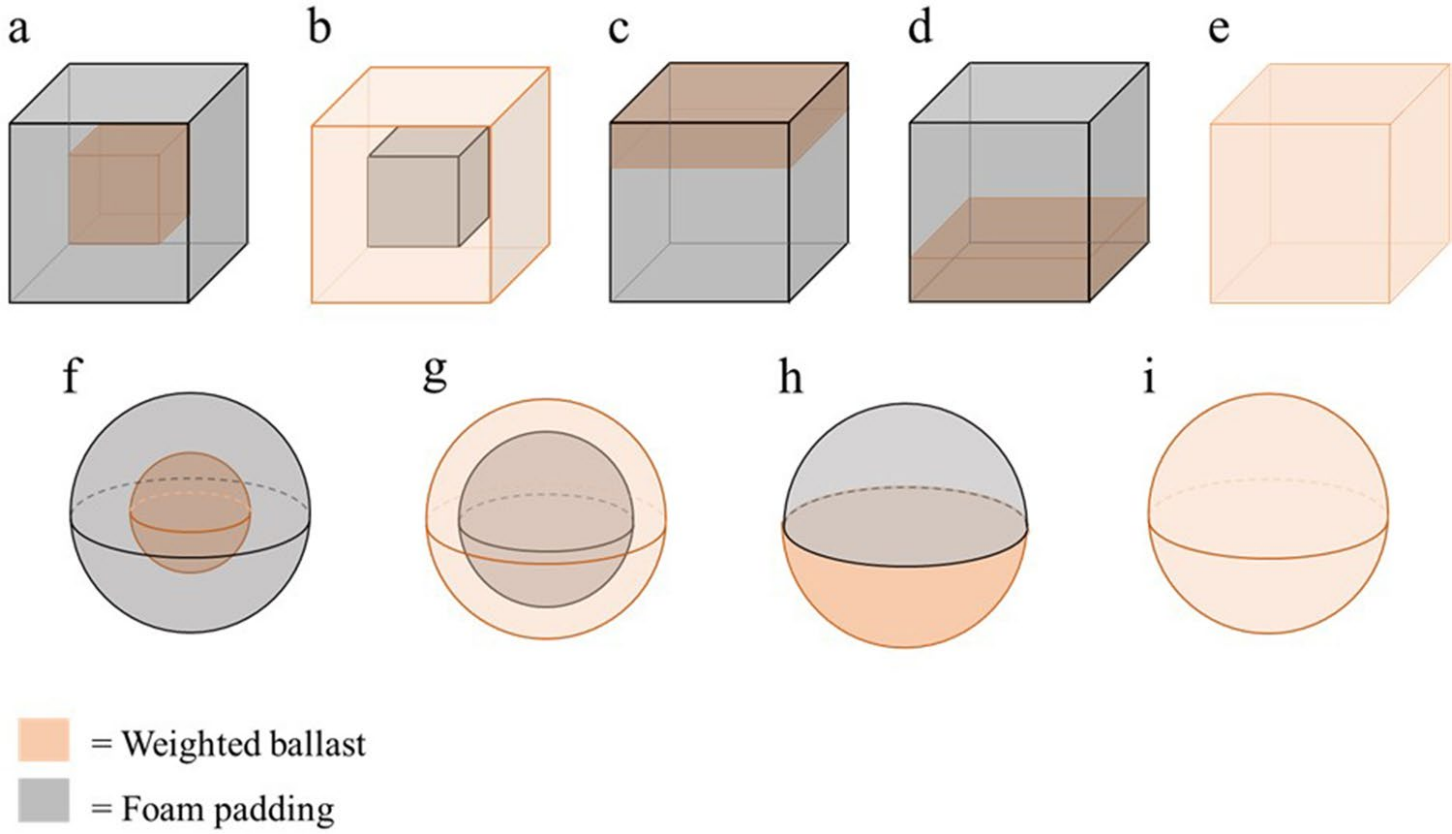
Mass distributions used within each set. Objects ‘**a**’ to ‘**e**’ show the five mass distributions used for the cubes. Objects ‘**f**’ to ‘**i**’ show the four mass distributions used for the spheres. Note, the same sphere (**h**) was used in both the “bottom” and “top” mass distributions by flipping the object. The orange area indicates where the weighted ballast was placed in the objects while the grey areas indicate the position of the foam

We used two sets of five three-dimensional (3D) printed hollow cubes that were painted black and were 6 × 6 × 6 cm with a volume of 216 cm^3^. We attempted to engineer them to each weigh 245.5g. Their actual weight after construction ranged from 245.5 g to 247.0 g (average = 245.95 g; Table 1), which included the cube shell itself, weighted ballast and foam padding. Note, this range is minute, representing a 0.006 ﻿× difference (approximately a third of a sheet of A4 paper), and falls well below the threshold of a just noticeable difference (Weber, 1831; Wolf & Drewing, 2020), meaning differences could be considered imperceptible. As the cubes were the same size and had similar masses, their density was equally similar, ranging from 1.137 g/cm^3^ to 1.144 g/cm^3^ (average = 1.139 g/cm^3^). Within each set, one cube had its mass positioned at the “top” and another had its mass positioned at the “bottom”. There was also a cube with the mass distributed along its six faces (“outside”), one with the mass positioned internally in the geometric centre (“inside”), and finally, one with an “even” mass distribution (see Fig. 2). Each of the cubes, except for the one that had an evenly distributed mass, were weighted with lead shot placed between foam. The cube with an evenly distributed mass was filled with salt instead. We opted to use salt as a ballast as it achieved the desired mass with one material that filled the cube entirely. In one set of cubes, there was a small clip affixed to the top, which a handle or string could be attached. The other set of cubes remained flush.

**Table 1:** Stimuli masses

Lifting approach	Inside	Outside	Top	Bottom	Even
Cube	245.5	245.5	245.3	245.6	245.5
Handle/string	247	245.6	246.1	246.1	246.8
Sphere	247.1	247.7	248	248	247.8

All masses are in grams. For the Sphere, the “Top” and “Bottom” mass distributions used the same object, just rotated

In addition to the cubes, there were four 3D printed hollow spheres. Although we attempted to match them in terms of both volume (216 cm^3^) and mass (245.5 g), there were again minute differences in construction. The spheres had a circumference of 23.5 cm and a volume of 219 cm^3^, representing 3 cm^3^ more volume than the cubes, or approximately a 1% difference. Their actual weight after construction ranged from 247.1 g to 248 g (average = 247.8 g, representing a 0.007 ﻿× difference compared with the cubes; Table 1) with a density ranging from 1.128 g/cm^3^ to 1.132 g/cm^3^ (average = 1.132 g/cm^3^, representing a 0.006 × difference compared with the cubes). The spheres were filled in an identical manner to the cubes. The same sphere was used in both the “top” and “bottom” mass distribution trials, it was just rotated. Hence four spheres were used (see Fig. 2). The same was not done with the cubes for two reasons. Firstly, the bottom of the cubes had a noticeable seam outlining the bottom face, which was removable to allow filling. Secondly, even if we disguised the seams, we would not have been able to flip and place some of our cubes on a surface because of the clips on top.

Note, although very slight variations existed in mass, volume and density among the cubes and spheres, these differences in construction were again considered perceptually negligible. Moreover, although previous studies have suggested that cubes might be visually perceived as larger than equivalent spheres (Kahrimanovic et al., 2010), it is unlikely for this small visual discrepancy to lead to much change in weight perception when haptic information is available. Visual information does not contribute to the size-weight illusion nearly to the same extent as haptic information (Ellis & Lederman, 1993).

These five mass distributions imparted different rotational dynamics. The “inside” objects had a lower rotational inertia compared to the “even” objects, which had a lower rotational inertia compared to the “outside” objects. This is because comparatively more mass was moved over the same distance when rotation occurred, meaning more energy would have been required to initiate displacement as it had a greater resistance to being rotated. With regard to the “top” and “bottom” objects, they were both more asymmetrical than the other three mass distributions. However, the “top” objects had their mass positioned more towards the point of rotation (i.e., they were closer to the wrist) and would have had a lower rotational inertia compared to the “bottom” objects. Finally, as compared to their cube counter parts, the spheres were more rotationally symmetrical given their shape, and were therefore predicted to be perceived as lighter.

The manner with which participants lifted the objects was also manipulated. In one, the cubes were lifted with a handle with a pinching motion between the index finger and thumb. In another, the cubes were lifted from a ring on a 14-cm long string that was attached to the cubes. The last two lifting conditions allowed participants to use their entire hand to lift the spheres and clipless cubes directly. The above lifting conditions are referred to as Handle, String, Sphere and Cube, respectively. The objects lifted directly – the clipless cubes and spheres – allowed participants the best opportunity to detect differences in rotational dynamics as compared to the handles, which themselves conveyed more rotational information than the strings.

The height of the surface on which the objects was placed was controlled for such that lifting occurred from the same height. In the direct lifting condition, the cubes were placed on a slight platform so they were at the same height as when the cubes had a handle attached to them (i.e., the top surface of the cube matched the top of the handle). For the string condition, the cubes were placed on a lower surface, such that when the string had no slack, it too was at the same height as the top of the handle. In doing this, we ensured that there would be no differences in the joint along which rotation occurred and that rotational dynamics would not differ due to the starting location of the joints and consequently the axis of rotation.

To help eliminate response biases (Firestone & Scholl, 2016), participants were equipped with a pair of PLATO Visual Occlusion Spectacles (Translucent Technologies Inc., Toronto, Canada) that remained closed between trials, preventing them from generating expectations of the stimuli’s weight by observing the experimenter interacting with them. To further safeguard against such extraneous variables, stimuli that were not currently being used in a trial were hidden behind a screen so they were out of view when the shutter goggles were open. There was also a tactile marker on the table 10 cm away from the stimuli, which could be found without vision, which allowed participants to position their hand in the same starting location prior to every trial. Participants were instructed to lift the objects in a manner that felt natural to them, but to also maintain consistency throughout the experiment. Additionally, as part of the instructions, participants were told that there were no right or wrong answers and that they should report the object’s weight as they truly perceive it, not as they think it may or should weigh.

A trial started when the shutter goggles opened, at which time the participant gripped the object and lifted it off the surface it was resting on and then placed it back down when the shutter goggles closed 5 s after they opened. Participants gave their perceptual estimates of the object’s weight at any point during the trial after the lift occurred. Within each lifting condition, each mass distribution was lifted 15 times in a block for a total of 300 lifts across the experiment. The order of the conditions was randomised as was the order in which each cube was lifted within each condition.

To acquire perceptual estimates, we used absolute magnitude estimates (Zwislocki & Goodman, 1980) in which participants ascribed a number to the objects that best encapsulated their apparent weight, where greater numbers were associated with a greater perceived weight. There were no limits on the numerical scale that participants used for their magnitude estimates. However, they were asked to remain consistent with their chosen scale throughout the experiment.

#### Experiment 2

In this experiment, we tested if the participants could correctly identify how the mass was distributed within the objects. After the participants completed all 300 lifts, they were presented with all five clipless cubes at the same time, which were randomly ordered from left to right, and a sheet of paper that had the five mass distributions, as shown in Fig. 2, printed on it. Participants were asked to lift the cubes and place them on the image on the sheet of paper that corresponded to the mass distribution they believed was present in each object. Participants were allowed as much time as they wanted to complete this task and were allowed to freely interact with each object but were asked not to flip them upside down. A similar procedure was used for the four spheres. However, for every participant, the sphere that was used for the “top” and “bottom” mass distributions was presented to them in only its “bottom” orientation (i.e., having its mass on the bottom of the sphere). These perceived judgements in mass distribution were recorded.

### Data preparation and analysis

We converted the participants’ perceptual magnitude estimates into percentage scores to normalise them to the same scale across participants. Specifically, for each participant, we subtracted their individual scores from their total estimate mean, then divided that number by the total average estimate before multiplying the resulting number by 100. The resulting score indicates a percentage difference from the overall mean. For each participant, we then took the mean value of their lifts within each condition, which was then used in our final analysis. Regarding the mass distribution reports, the participant’s reports were simply coded as either correct (1) or incorrect (0).

Statistical analyses were done using JASP (Version 0.16.4, University of Amsterdam, Amsterdam, The Netherlands) and GraphPad Prism (Version 9.1, GraphPad Software, San Diego, CA, USA). Where appropriate, Greenhouse-Geisser corrections were made for violations of sphericity, as determined by Mauchly’s test of sphericity. Mean %-scores were analysed with a 4 (Handle, String, Cube, Sphere) × 5 (“inside”, “outside”, “top”, “bottom”, “even”) repeated-measures ANOVA to examine how the participant’s weight perception of the stimuli was influenced by their lifting approach and the object’s mass distribution. Tukey’s Honest Significant Difference (HSD) post hoc comparison tests were performed to further examine significant interactions and main effects (Tukey, 1949). Subjective mass distribution reports were analysed with one-sample t-tests against the lower limit chance value, i.e., > .25 for spheres and > .20 for cubes to determine if participants were more likely than chance at correctly judging the mass distribution of the cube and sphere stimuli.

## Results

### Overview

To summarise, in line with predictions, the first experiment found a significant interaction, indicating that the Sphere with the “inside” mass distribution was felt to be particularly light compared to the other stimuli and other significant comparisons. There was also a significant main effect of Lifting Approach where stimuli lifted by the Strings were perceived to be heavier than each of the other lifting approaches. A significant main effect of Mass Distribution was also found. This revealed that objects with the “inside” mass distribution were perceived to be lighter than those with the “bottom” and “even” distributions, and those with the “outside” distribution were also felt to be lighter than those with the “bottom” distribution. Experiment 2 revealed that participants were significantly more likely than chance at correctly identifying only the Sphere with the “bottom” mass distribution. Next, we provide more detailed statistics supporting these findings.

### Perceived weight experiment: Lifting approach × mass distribution interaction

**Fig. 3:**
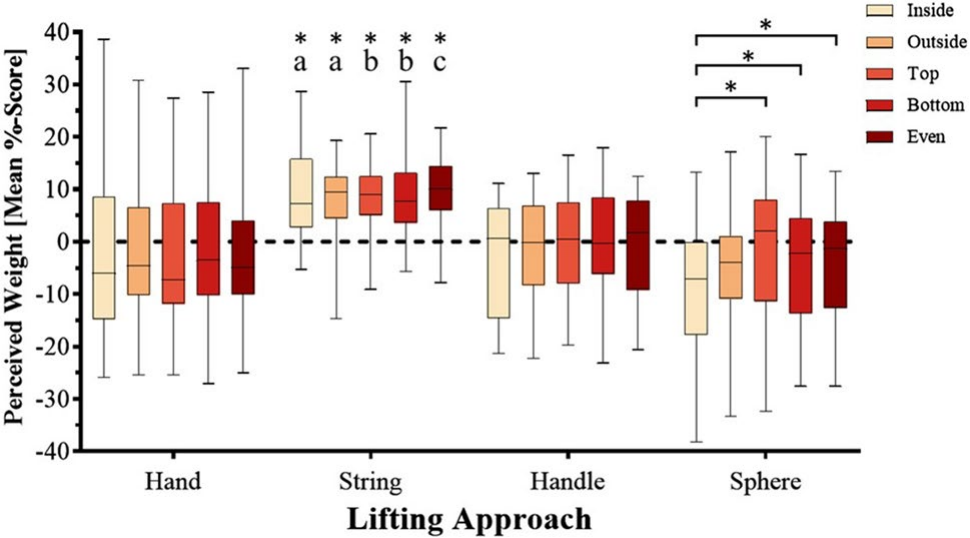
Perceived weight (mean %-score) of objects by lifting approach and mass distribution. For each box, the whiskers represent the minimum and maximum range while the box proper represents the middle 50% of the data, 25% above the median line, and 25% below. Asterisks (*) denote significant differences after Tukey corrections were applied (*p* < .05). Letter-labelled asterisks (*) denote differences from (**a**): the Sphere with the “inside” mass distribution; (**b**) the Spheres with the “inside”, “outside”, and “even” mass distributions; and (**c**) the Spheres with the “inside”, “outside”, “bottom”, and “even” mass distributions.

There was a significant Lifting Approach × Mass Distribution interaction, F(6 ,114.6) = 2.6, *p* = .02, η^2^_p_ = .12 (see Fig. 3). Post hoc testing revealed that the Sphere with the “inside” mass distribution was felt to be lighter than the Spheres with the “top”, “bottom” and “even” mass distributions with a mean perceptual magnitude %-score difference between 4.5 and 7.6. It was also perceived as lighter than the String stimuli with the “inside”, “outside”, “top”, “bottom” and “even” mass distributions with a mean perceptual %-score difference between 15.6 and 18. The Spheres with the “outside” and “even” mass distribution were perceived as lighter than the String stimuli with the “top”, “bottom” and “even” distributions, with mean %-score difference ranging between 11.9 and 14.2. Finally, the Sphere with the “bottom” mass distribution was also perceived as being 13.4% lighter than the String stimuli with the “even” distribution. See Table 2. for a summary of all significant interaction effects. All other comparisons were non-significant (all *p*s > .05).

**Table 2.** Significant lifting approach by mass distribution post hoc comparisons for mean %-scores

Comparison		Mean		Mean diff.	*p*	*d*
	String inside		9	17.5	.003**	1.66
	String outside		7.1	15.6	.01*	1.52
	String top		8.4	16.9	.002**	1.78
Sphere inside	String bottom	-8.5	9.5	18	.003**	1.72
	String even		9.4	17.9	.001**	1.88
	Sphere top		-0.9	7.6	.01*	0.6
	Sphere bottom		-4	4.5	.02*	0.4
	Sphere even		-3.5	5	.03*	0.45
	String top		8.4	13.1	.02*	1.39
Sphere outside	String bottom	-4.7	9.5	14.2	.04*	1.37
	String even		9.4	14.1	.02*	1.49
	String top		8.4	11.9	.04*	1.27
Sphere even	String bottom	-3.5	9.5	13	.04*	1.26
	String even		9.4	12.9	.03*	1.38
Sphere bottom	String even	-4	9.4	13.4	.04*	1.4

Corrected using Tukey’s HSD. Asterisks (*) denote significant differences. * *p* < .05, ** *p* < .01

### Perceived weight experiment: Main effect of lifting approach

**Fig. 4:**
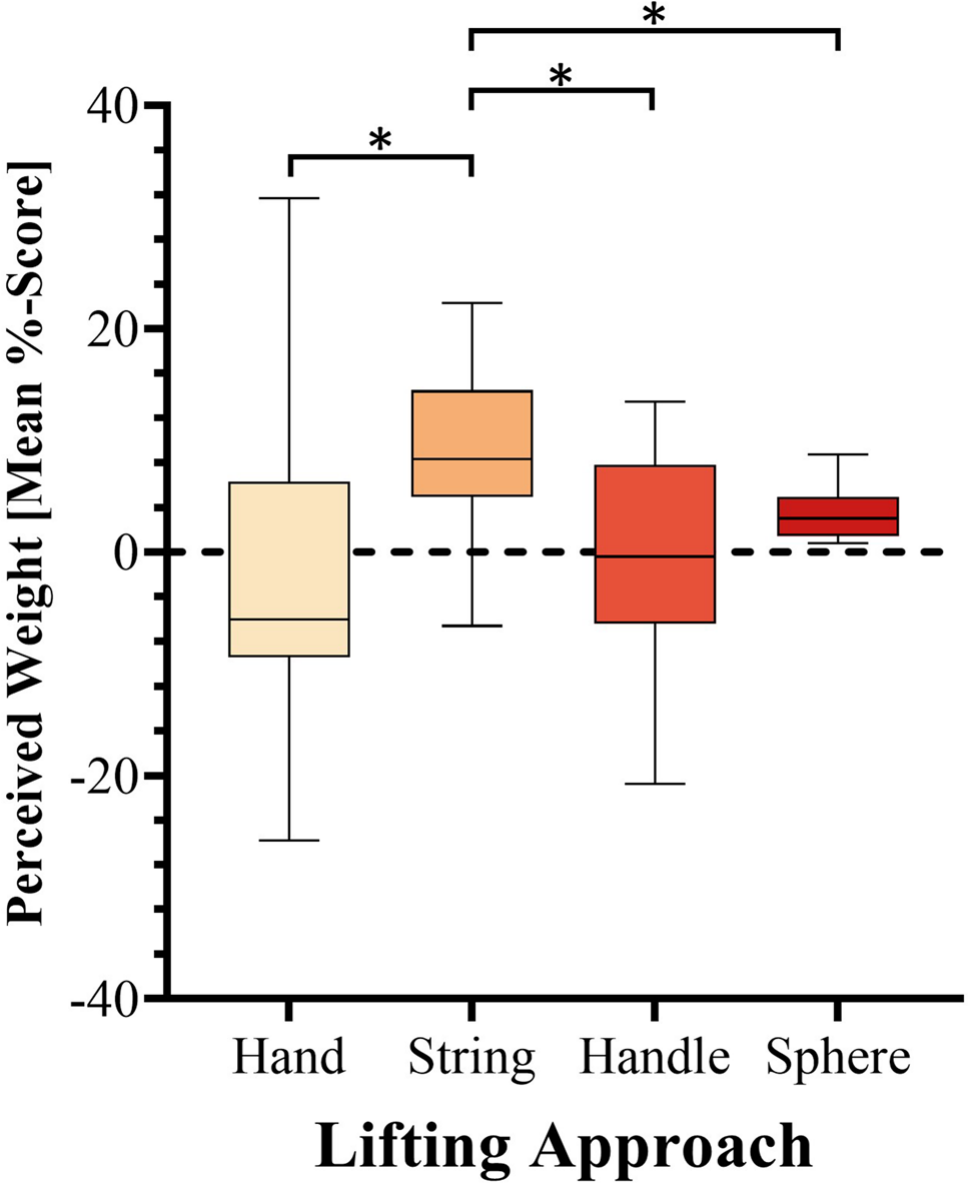
Perceived weight (mean %-score) of objects by lifting approach. For each box, the whiskers represent the minimum and maximum range while the box proper represents the middle 50% of the data, 25% above the median line, and 25% below. Asterisks (*) denote significant differences. * *p* < .05

ANOVA revealed a significant main effect of Lifting Approach, F(3, 57) = 4.6, *p* = .006, η^2^_p_ = .2 (see Fig. 4). Post hoc testing found that the stimuli lifted via the Strings (mean = 8.7) were perceived as heavier than each of the other lifting approaches with a mean %-score difference of 10 for the Handles (mean = -1.3, *p* = .03, *d* = 1.1), 11.8 for those lifted directly with the Hand (mean = -3.1, *p* = .03, *d* = 1.1), and 13 as compared to the Sphere (mean = -4.3, *p* = .003, *d* = 1.4). In other words, objects lifted by the string were perceived as being uniquely heavy as compared to the lifting methods. All other comparisons were non-significant (*p* > .05).

### Perceived weight experiment: Main effect of mass distribution

**Fig. 5:**
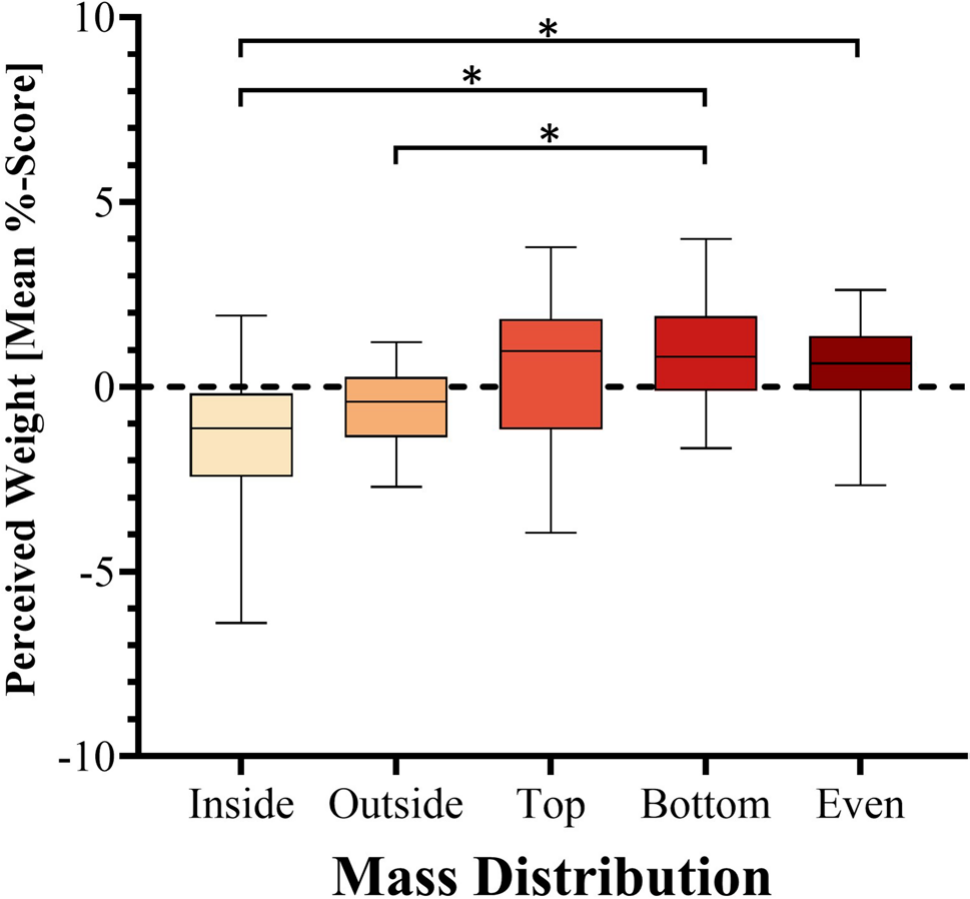
Perceived weight (mean %-score) of objects by mass distribution. For each box, the whiskers represent the minimum and maximum range while the box proper represents the middle 50% of the data, 25% above the median line, and 25% below. Asterisks (*) denote significant differences. * *p* < .05

ANOVA also revealed a significant main effect of Mass Distribution, F(2.6, 48.8) = 5.5, *p* = .004, η^2^_p_ = .22 (see Fig. 5). Post hoc testing revealed that the objects with the “inside” mass distribution (mean = -1.4) were perceived as being lighter than those with the “bottom” (mean = 0.5) and “even” (mean = 0.5) distributions, with a %-score mean difference of 2.2 (*p* = .04, *d* = 1.3), and 1.9 (*p* = .01, *d* = 1.1), respectively. Objects with the “outside” mass distribution (mean = -0.6) were also found to be lighter than those with the “bottom” mass distribution (mean = 0.9), with a %-score mean difference of -1.4 (*p* = .01, *d* = 1.1). All other mass distribution differences were non-significant (all *p* > .05).

### Perceived mass distribution experiment

**Fig. 6:**
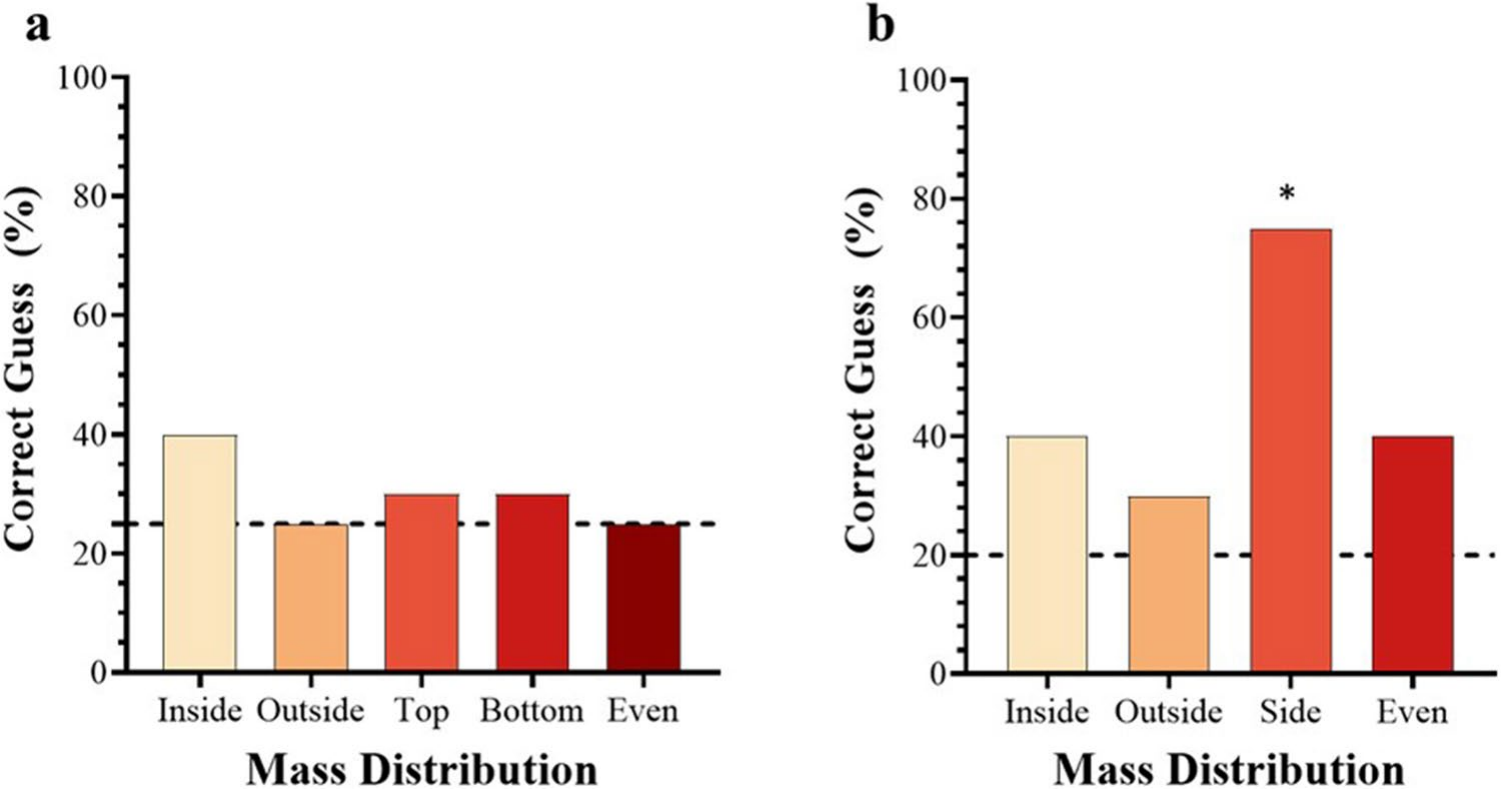
Correct judgements (%) of mass distribution for cubes and spheres. Subfigure ‘**a**’ regards the Cubes while subfigure ‘**b**’ regards the Spheres. The dashed lines represent the chance values (.2 for the Cubes and .25 for the Spheres). The asterisk (*) denotes a significant difference. * *p* < .05

Regarding participants’ reports of perceived mass distribution (see Fig. 6), out of the spheres, only the one with the mass distributed entirely on one side (i.e., the “top”/“bottom” sphere) was judged correctly at greater than chance levels (*t*(19) = 5, *p* < .001, *d* = 1.1, for a test value of > .25), having been identified by 79% of participants. As for the cubes, none of the mass distributions were correctly identified by participants at above chance levels (all *p*s > .05 for a test value of > .2).

## Discussion

We aimed to determine if the predictions of the rotational dynamics account of weight perception are evident with more naturalistic objects lifted in a more ecological manner than those used in earlier studies. In doing so, we manipulated the rotational dynamics and rotational symmetry of cubes and spheres, as well as our ability to detect them. Overall, the effects we found were in line with those predictions.

As highlighted in the *Introduction*, the rotational dynamics account of weight perception (Amazeen & Turvey, 1996; Carello & Turvey, 2000) makes several clear predictions. Firstly, it predicts that the perception of object weight is related to their rotational dynamics. The more resistance an object has to rotation, the heavier it will feel. Secondly, it predicts that as objects become more symmetric in their rotational dynamics as a result of their mass distribution, the lighter they will feel. Finally, it predicts that as weight perception is based on rotational dynamics, its influence will be related to our ability to detect those forces. Namely, the harder they are to detect, the less influence they will have. Each one of these predictions was apparent in the results obtained.

Out of all possible factor combinations, the lightest perceived object was the one constructed with the mass distribution associated with the lowest rotational inertia – in the geometric centre of the object – in a shape that was the most symmetrical, being a sphere. Indeed, the sphere with the “inside” mass distribution accounted for over half of all the significant interaction comparisons. Additionally, it was significantly lighter than all the objects lifted with the strings, meaning that when the rotational dynamics of the objects were removed, their perceived weight increased. This was further supported by the main effect we found for the lifting approach: those objects lifted with strings were uniquely heavy as compared to those other approaches.

Even though rotational dynamics affected weight perception, there was limited evidence to suggest that its awareness had an impact. In Experiment 2, participants could only discern the mass distribution of the sphere with its mass distributed on one side. This is interesting as how the mass was distributed in the object did influence how heavy it was perceived to be, as demonstrated by the main effect of mass distribution. This would suggest that awareness of subtle differences in rotational dynamics is not required for rotational dynamics to impact weight perception. The effects of rotational dynamics seem to occur outside of conscious awareness.

Importantly for the rotational dynamics theory, our results confirm that weight perception can also change in a manner predicted by this theory when objects are more naturalistic and when rotational dynamics are not uncannily exaggerated. This lends support to the notion that rotational inertia is a broadly applicable physical influence contributing to the perception of object weights, rather than being relegated to an effect that only occurs under specific, artificial and favourable conditions.

For example, as compared to differences in tensor objects, the differences in rotational dynamics between the stimuli we used would have been more subtle given that our stimuli were lighter and had less volume, which was reflected in their differences in mass distribution. Additionally, while there would have been some bodily rotation during the participants’ lifts, for example, in the wrist, elbow and shoulder, the natural lifting behaviour would have caused less bodily rotation as compared to previous studies using tensor objects, conceivably reducing the participant’s ability to feel it. Indeed, Experiment 2 reveals that participants were unable to judge the correct mass distributions for most stimuli. Yet, the interaction we found still had a medium effect size, speaking to the influence of rotational dynamics even outside of conscious awareness, most likely mediated by some sort of bottom-up mechanism.

This suggestion highlights an interesting question for future research. Could a conscious awareness of differences in mass distribution amplify differences in weight perception, possibly in a top-down manner? While a matter more so for future research, this suggested difference has the potential to yield stronger effects. It is also worth noting that we used one consistent mass for all stimuli. Generalising our conclusions to objects with other masses requires further research.

Related to the above, previous research using non-tensor objects also examined the influence of modifying the mass distribution of objects on their perceived weight. For example, Koseleff (1937) shifted a large portion of an object’s overall weight 5 cm from the top to the bottom of a 7-cm tall object. Similarly, Paulun et al. (2019) shifted a smaller absolute and proportion of mass in their objects over a smaller distance. In both cases, the overall perceived weight of the object changed when the mass distribution was modified. Our stimuli were even more subtle, were lighter, had less volume, and the shifts in their mass distribution were smaller than those of two preceding studies, and yet we too found a difference in the perceived weight of our objects. Again, this is indicative, and supportive, of the hypothesised influence of rotation dynamics posited by Amazeen and Turvey (1996).

There are other interesting lessons to be had from our study. Within the size-weight illusion literature, for example, there is inconsistency in the method used to weight objects. Some authors indicate that they placed their weights in the geometric centre of the stimuli (Buckingham, 2019) while others state that weight was evenly, or approximately evenly, distributed within the objects by drilling holes in them (Flanagan et al., 2008; Saccone & Chouinard, 2019) or by uniformly filling putty with lead shot (Flanagan & Beltzner, 2000). The rotational dynamics explanation posits that these two main methods would result in different inertia and influence the perceived weight of objects differently. In the present study, we revealed that the “inside” sphere was perceived as heavier than the “even” sphere. It is worth considering that these effects were present in objects with the same size. It could be the case that the method used to weight objects can alter the degree to which differences in volume affect perceived weight, such as those observed in the size-weight illusion. Further research is required to examine this possibility.

Our results are difficult to reconcile with the mass-density account of weight perception (Wolf et al., 2018). The general thesis of this account is that weight perception derives from the average between an estimate about the object’s mass and an estimate about the object’s density, weighted based on their reliability. The interaction we had demonstrating a difference in perceived weight between spheres is difficult to be accounted for by this explanation. All spheres had the same density, the same mass, and the participant’s reliability to detect them were conceivably equal. Yet, differences in perceived weight were still observed between spheres. Perhaps the mass-density account of weight perception could still explain our results if rotational dynamics, for whatever reason, cause errors in how mass and density are computed or integrated. Alternatively, it is conceivable that objects that do not have a uniform density throughout might be processed slightly differently to those with a uniform density; however, more research would be required.

In closing, our study provides evidence for the rotational dynamics explanation of weight perception in the ecological handling of naturalistic objects. This allows for a more generalised application of the theory than what was demonstrated before. Our results also highlight that explanations of weight perception and weight illusions should account for rotational dynamics. The underpinning of weight perception and weight illusions is likely multifactorial, and we posit that rotational dynamics is as an important factor to consider as mass, volume, density and expectations.

## Data Availability

Data is publicly available on the Open Sciences Framework and can be found at the following url: https://osf.io/cuztb/

## Declarations

### Preregistration

The experiments here were not preregistered.
